# Differential Pulse Voltammetric Electrochemical Sensor for the Detection of Etidronic Acid in Pharmaceutical Samples by Using rGO-Ag@SiO_2_/Au PCB

**DOI:** 10.3390/nano10071368

**Published:** 2020-07-14

**Authors:** Sathish Panneer Selvam, Somasekhar R. Chinnadayyala, Sungbo Cho, Kyusik Yun

**Affiliations:** 1 Department of Electronics Engineering, Gachon University, Seongnam-si, Gyeonggi-do 13210, Korea; satp103@gc.gachon.ac.kr (S.P.S.); ssreddy@gachon.ac.kr (S.R.C.); 2 Gachon Advanced Institute for Health Science & Technology, Gachon University, Incheon 21999, Korea; 3 Department of Bionanotechnology, Gachon University, Seongnam-si, Gyeonggi-do 13210, Korea

**Keywords:** etidronic acid, differential pulse voltammetry, self-assembly, ultrasonic irradiation

## Abstract

An rGO-Ag@SiO_2_ nanocomposite-based electrochemical sensor was developed to detect etidronic acid (EA) using the differential pulse voltammetric (DPV) technique. Rapid self-assembly of the rGO-Ag@SiO_2_ nanocomposite was accomplished through probe sonication. The developed rGO-Ag@SiO_2_ nanocomposite was used as an electrochemical sensing platform by drop-casting on a gold (Au) printed circuit board (PCB). Cyclic voltammetry (CV) and electrochemical impedance spectroscopy (EIS) confirmed the enhanced electrochemical active surface area (ECASA) and low charge transfer resistance (R_ct_) of the rGO-Ag@SiO_2_/Au PCB. The accelerated electron transfer and the high number of active sites on the rGO-Ag@SiO_2_/Au PCB resulted in the electrochemical detection of EA through the DPV technique with a limit of detection (LOD) of 0.68 μM and a linear range of 2.0–200.0 μM. The constructed DPV sensor exhibited high selectivity toward EA, high reproducibility in terms of different Au PCBs, excellent repeatability, and long-term stability in storage at room temperature (25 °C). The real-time application of the rGO-Ag@SiO_2_/Au PCB for EA detection was investigated using EA-based pharmaceutical samples. Recovery percentages between 96.2% and 102.9% were obtained. The developed DPV sensor based on an rGO-Ag@SiO_2_/Au PCB could be used to detect other electrochemically active species following optimization under certain conditions.

## 1. Introduction

Bisphosphonate compounds are a category of drugs in the contemporary pharmacological arsenal that avert bone density damage. Etidronic acid (EA), which is also called hydroxyethylidene diphosphonic acid (HEDP), is a member of the bisphosphonate group. EA is used as an active ingredient for cosmetic agents, medication, water treatment, and chelating agents [1]. Etidronate has the lowest potency of all bisphosphonates, which causes high diffusional distribution through the bone surface. In medication, EA is mainly used to reduce osteoclastic bone resorption (Paget’s disease and osteoporosis), and only very low concentrations (200–400 mg) of etidronate are used to treat bone damage [2,3]. Etidronate has been approved in Canada and many European countries to treat osteoporosis. To treat symptomatic Paget’s disease, the US FDA approved etidronate, and thus it is widely used in the United States [4]. However, excess etidronate could lead to severe renal failure, joint pain, and low levels of calcium in the blood. The estimated overdose concentration of etidronate was found to be above 500 mg. The continuous accumulation of EA in the human body might also cause acute oral and dermal damage in humans [5]. Therefore, it is important to monitor the concentration of EA in real samples and pharmaceutical formulations [6].

Because of the absence of chromophoric and fluorophore agents, direct detection of EA through photometric and fluorometric estimation is impossible. The available conventional methods for determining EA involve direct determination by evaporative light-scattering detection and indirect detection through chemical derivatization. Chemical derivatization is time-consuming, and the resultant derivatives are at times unstable, making it difficult to carry out complete analysis [7]. The recently published United States Pharmacopeia defines the detection method of sodium etidronate as liquid chromatography assisted by a conductivity detector. The efficient design of the sensing platform, low sample volume consumption, and the lack of need of further pretreatment of the samples in the electrochemical sensors are the main factors in contemporary quantitative analysis [8]. Although conventional methods have been developed to detect EA, sensors with higher sensitivities and stabilities have not yet been achieved.

Electrochemical sensors possess nanomaterial-modified electrodes for enhancing the catalytic activity for analyte detection [9]. Enhancement of the electrocatalytic activity relies on the active surface area of the nanomaterials and the number of active sites in the nanomaterials [10,11]. Nanomaterials based on graphene and its derivatives possess excellent conductivity, high thermal stability, and inexpensive functionalization through chemical processes. For these reasons, a wide variety of electrochemistry-based studies on graphene-based nanomaterials have been carried out [12,13,14]. Electrochemical characteristic studies have confirmed the enhanced electron transport behavior of reduced graphene oxide (rGO), and thus it has been widely used as an electrochemical sensing platform. The vital role of rGO lies in increasing the number of adsorptive sites, which enhances the electrocatalytic ability of the rGO-modified electrodes [15,16,17].

Metal nanoparticles have been predominantly utilized in the construction of sensing platforms in electrochemical sensors [11,18,19]. Because metal nanoparticles possess a greater surface area-to-volume ratio, they are adsorbed onto graphene-like sheets to form novel nanocomposites to achieve excellent electrochemical performance. The adsorbed metal nanoparticles could be responsible for the improved electron transfer at the electrode interface, resulting in the excellent conductivities of these electrodes [20,21]. Silver nanoparticles are most frequently used with nanosheet structures to enhance the electrochemical performance of electrochemical sensors [22,23]. In this study, probe sonication-assisted construction was used to develop an rGO-Ag@SiO_2_ nanocomposite as an electrochemical sensing platform. This is the first differential pulse voltammetric (DPV)-based electrochemical sensor for the electrochemical detection of EA.

## 2. Experimental

### 2.1. Chemicals and Apparatus

Refer to the supplementary information for details about the chemicals and apparatus.

### 2.2. Synthesis of Ag@SiO_2_

An ultrasonication-assisted method was used to prepare Ag@SiO_2_ nanoparticles. Solution-A containing 7.5 wt.% tetraethyl orthosilicate (TEOS) in ethanol was injected into a solution containing 4.25 wt.% NH_4_OH in ethanol through a syringe pump at a flow rate of 0.8 mL/min. Then, the solution mixture was ultrasonicated at 30% amplitude for 30 min under a probe sonication temperature of 65 °C. To the above solution, an aqueous mixture containing 4.75 wt.% AgNO_3_ and 4.25 wt.% NaBH_4_ was injected dropwise, and the solution was ultrasonicated for 30 min. The as-prepared solution was centrifuged, washed twice with ethanol, and dried at 80 °C for 3 h [24].

### 2.3. Preparation of rGO

A modified Hummers method was used to prepare rGO. Initially, 1.0 g of graphite powder was dispersed in 40 mL of H_2_SO_4_ in a round-bottom flask, and the solution was kept in an ice bath. Next, 3.5 g of KMnO_4_ was added to the above-prepared solution and the reaction mixture was stirred for 2 h, followed by the addition of 150 mL of Milli-Q water. The dropwise addition of H_2_O_2_ was carried out when the reaction mixture no longer formed bubbles. The prepared solution was subsequently filtered to remove undesirable metal ions, and the obtained solution was washed with 10% HCl and completely dried. The prepared graphene oxide (GO) was dispersed in Milli-Q water, and under continuous stirring for 1 h at 80 °C, the reduction of GO to rGO was maintained by the introduction of 1 mL of hydrazine hydrate. The color of the obtained solution color changed to black, which confirmed the formation of rGO. The collected precipitate was filtered and subsequently dried in a vacuum oven [8].

### 2.4. Construction of the rGO-Ag@SiO_2_ PCB Sensing Platform

#### 2.4.1. Self-Assembly of rGO-Ag@SiO_2_

One milligram (1 mg) each of rGO and Ag@SiO_2_ (Figure S4a,b) were dispersed in 1 mL of Milli-Q water and probe sonicated for 5 min at 45% amplitude.

#### 2.4.2. Surface Modification of the Au PCB

An integrated circuit-based Au PCB was used as an electrode. The Au PCB comprised a working electrode with a diameter of 1.48 mm, a counter electrode 4.3 mm long and 1.6 mm wide, and a reference electrode 4.1 mm long and 0.8 mm wide (Figure S1). The Au PCB was sequentially washed with acetone, ethanol, and Milli-Q water. After the Au PCB was cleaned and dried, plasma treatment was applied to the Au PCB. Approximately 5 μL of the as-prepared rGO-Ag@SiO_2_ nanocomposite was drop-casted on the cleaned Au PCB [25].

### 2.5. Real Sample Preparation

Etidronate drug tablets (200 mg) were used to prepare three different concentrations (25.0, 50.0, and 100.0 μM) of etidronate real samples in a 0.1 M NaOH diluent. The tablets were finely ground with the help of a mortar and pestle and dispersed in a 0.1 M NaOH electrolyte as a diluent. The samples were then filtered to remove undissolved excipients. The detergent samples were diluted 100 times with 0.1 M NaOH to fit the peak current response of EA into the calibration curve. In the final calculation, the obtained concentration of EA was multiplied by a dilution factor.

## 3. Results and Discussion

### 3.1. Characterization

**Figure 1:**
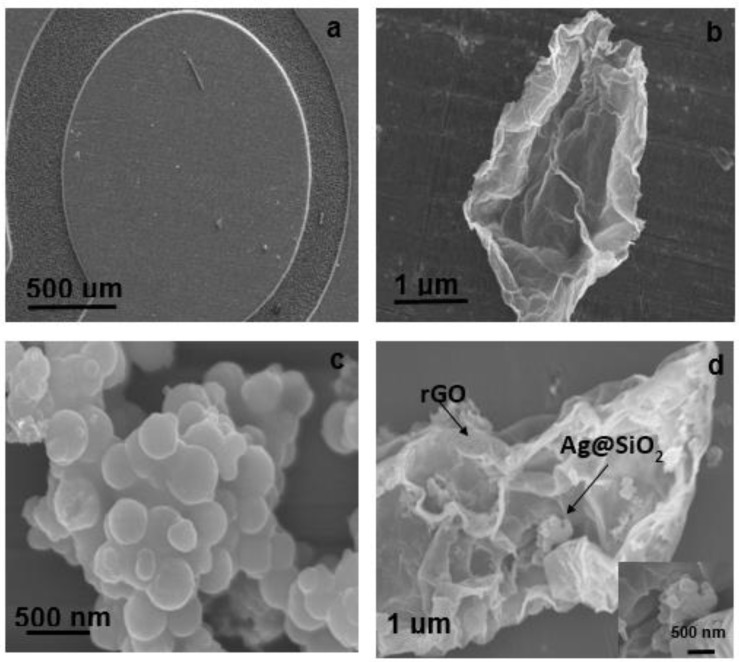
Scanning electron microscopy (SEM) images of (**a**) bare Au PCB, (**b**) rGO/Au PCB, (**c**) Ag@SiO_2_/Au PCB, and (**d**) rGO- Ag@SiO_2_/Au PCB.

The surface morphologies of the bare Au PCB, rGO-modified Au PCB, Ag@SiO_2_-modified Au PCB, and rGO-Ag@SiO_2_–modified Au PCB were examined by scanning electron microscopy (SEM). A smooth surface was observed for the bare Au PCB (Figure 1a), which turned into a thin sheet-like monolayer structure at the surface of the Au PCB owing to rGO (Figure 1b). The Ag@SiO_2_-modified Au PCB showed homogenous, evenly distributed Ag@SiO_2_ microspheres (Figure 1c). Examination of the rGO-Ag@SiO_2_–modified Au PCB electrode revealed the successful adsorption of Ag@SiO_2_ microspheres onto the rGO monolayer (Figure 1d).

**Figure 2:**
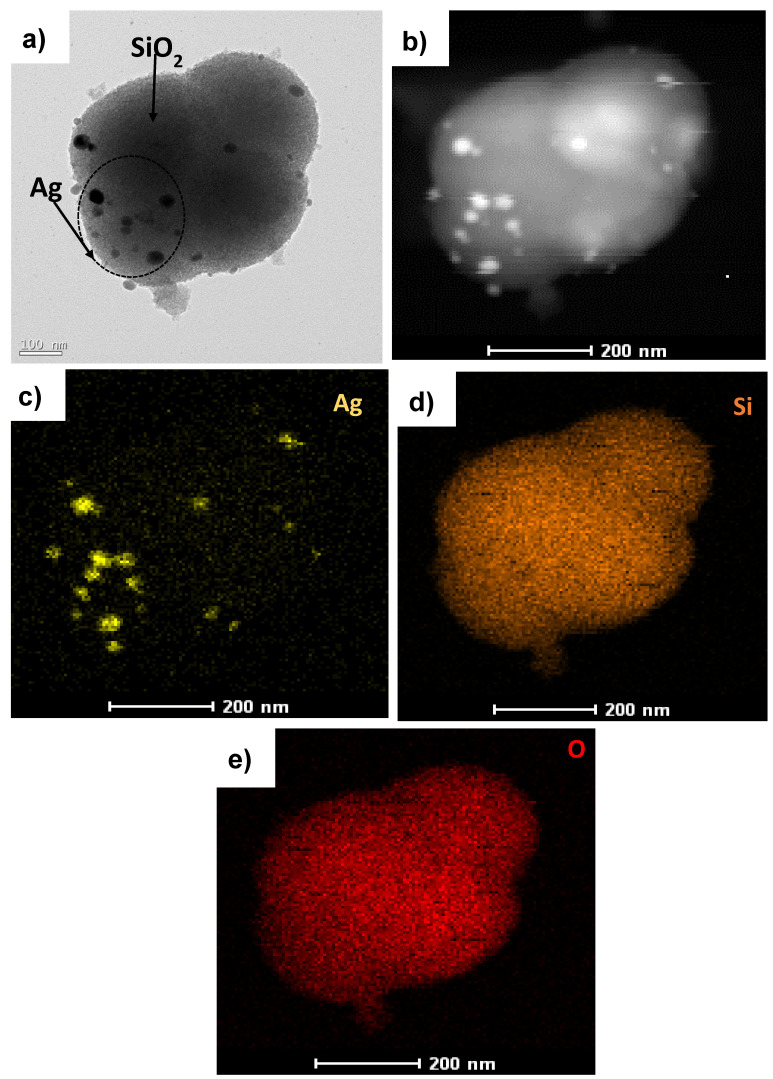
(**a**) Transmission electron microscopy (TEM) image of Ag@SiO_2_, (**b**) high-angle annular dark-field (HAADF) image of Ag@SiO_2_, and EDX mapping of (**c**) Ag, (**d**) Si, and (**e**) O.

To recognize the Ag-embedded SiO_2_ nanoparticles, TEM analysis was carried out, and Figure 2a shows the Ag@SiO_2_ nanostructure. The image confirms the presence of spherical SiO_2_ nanoparticles, with Ag nanoparticles embedded on the surface of the SiO_2_. Figure 2b shows the high-angle annular dark-field (HAADF) image of Ag@SiO_2_. The elements in Ag@SiO_2_ were classified by energy-dispersive X-ray spectroscopy (EDX) mapping, which confirmed the presence of a spatial distribution of elements such as Ag (Figure 2c), Si (Figure 2d), and O (Figure 2e) in Ag@SiO_2_.

The elemental compositions of the Au PCB, rGO/Au PCB, and rGO-Ag@SiO_2_/Au PCB were analyzed through EDX spectral analysis. The predominant elements observed at the Au PCB were Ni, Au, C, and O, with corresponding weight percentages of 63.4 wt.%, 30.7 wt.%, 5.2 wt.%, and 0.7 wt.% (Figure S2a). The rGO-modified Au PCB contained 57.9 wt.% C and 4.9 wt.% O (Figure S2b). EDX analysis of the rGO-Ag@SiO_2_/Au PCB confirmed the presence of C, O, Ag, and Si, with corresponding weight percentages of 16.0 wt.%, 8.8 wt.%, 48.6 wt.%, and 1.4 wt.% (Figure S2c).

**Figure 3:**
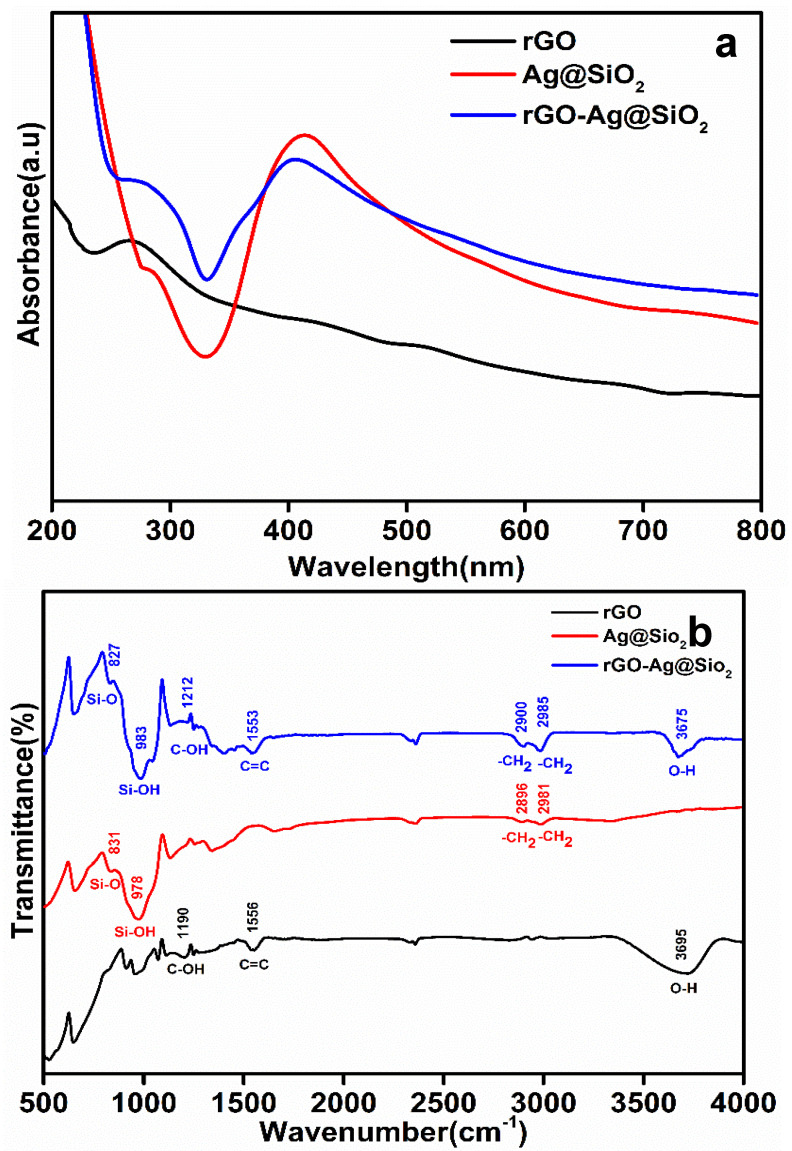
(**a**) The UV absorption spectra and (**b**) Fourier transform infrared (FTIR) spectra of rGO, Ag@SiO_2_, and rGO-Ag@SiO_2_.

Absorption spectral analyses of rGO, Ag@SiO_2_, and rGO-Ag@SiO_2_ were carried out in the wavelength range of 200–800 nm (Figure 3a).

An absorption peak of rGO was observed at 272.7 nm, and a highly intense absorption peak of Ag@SiO_2_ was observed at 410.2 nm [26,27]. The rGO-Ag@SiO_2_ nanocomposite showed absorption peaks for rGO and Ag@SiO_2_ at wavelengths of 276.8 and 408.6 nm, respectively. The presence of the two different absorption peaks for the rGO-Ag@SiO_2_ nanocomposite proved the presence of rGO and Ag@SiO_2_.

The individual functional groups of rGO, Ag@SiO_2_, and rGO-Ag@SiO_2_ were studied by Fourier transform infrared (FTIR) spectroscopy. The analysis was carried out using wavenumbers from 400 to 4000 cm^−1^ (Figure 3b). C–OH (1190 cm^−1^), C=C (1556 cm^−1^), and O–H (3695 cm^−1^) vibrations were identified in rGO. The bands recorded in Ag@SiO_2_ at wavenumbers of 831, 978, 2896, and 2981 cm^−1^ corresponded to Si–O, Si–OH, –CH_2_, and –CH_2_, respectively. After the rGO-Ag@SiO_2_ nanocomposite was formed, the individual vibrations present in the rGO and Ag@SiO_2_ were confirmed to be the C–OH (1212 cm^−1^), C=C (1553 cm^−1^), and O–H (3675 cm^−1^) vibration bands of rGO and the Si–O (827 cm^−1^), Si–OH (983 cm^−1^), –CH_2_ (2900 cm^−1^), and –CH_2_ (2985 cm^−1^) bands of Ag@SiO_2_. The presence of the individual bands of rGO and Ag@SiO_2_ at rGO-Ag@SiO_2_ proved that the nanocomposite was successfully formed owing to a simple probe sonication process [27].

**Figure 4:**
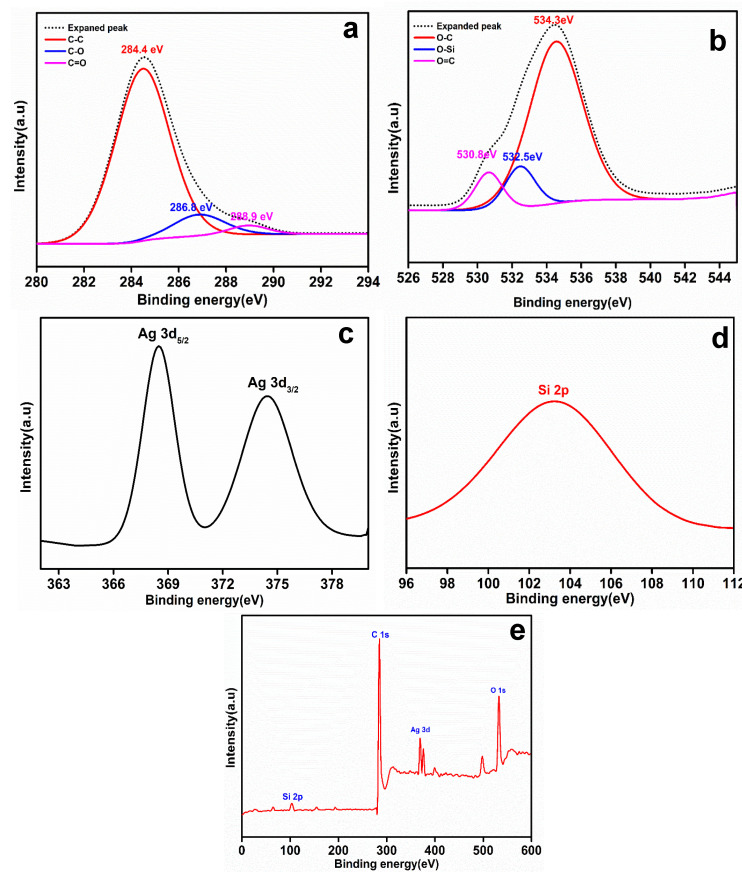
X-ray photoelectron spectroscopy (XPS) spectrum of rGO-Ag@SiO_2_. (**a**) Deconvoluted C 1S spectrum, (**b**) deconvoluted O 1S spectrum, (**c**) expanded Ag 3d spectrum, (**d**) expanded Si 2p spectrum of rGO-Ag@SiO_2_, and (**e**) full survey.

X-ray photoelectron spectroscopy was used to analyze the elements and their electronic states in the rGO-Ag@SiO_2_ nanocomposite. The overall survey (Figure 4e) showed the presence of C, O, Ag, and Si in the rGO-Ag@SiO_2_ nanocomposite. In the expanded C 1S spectrum (Figure 4a), the deconvoluted peaks were at 284.4, 286.8, and 288.9 eV, attributed to the C–C, C–O, and C=O spectra, respectively [28]. The expanded O 1s spectrum (Figure 4b) showed deconvoluted peaks corresponding to the C–O, Si–O, and C=O spectra at 534.3, 532.5, and 530.8 eV, respectively [29,30]. The expanded Ag 3d spectrum, shown in Figure 4c, contained two orbitals attributed to Ag 3d_5/2_ (368.5 eV) and Ag 3d_3/2_ (374.4 eV). An expanded Si 2p spectrum (Figure 4d) was observed at 103.2 eV [31].

### 3.2. Electrochemical Characterization of rGO-Ag@SiO_2_/Au PCB

Two methods were used to estimate the electrochemical active surface area (ECASA). The first method involved the estimation of the roughness factor, and the second method involved the evaluation of ECASA in the presence of a redox probe [(K_3_Fe(CN)_6_]. The roughness factor of the electrode (R_f_) was calculated from the double-layer capacitance (*C*_dl_). The non-faradaic current was captured at different scan rates (25 to 200 mV/s) with a potential window from −0.7 to −0.2 V. The current density at −0.45 V was considered to construct the *C*_dl_ [32]. Figure S3a,b show the cyclic voltammetry (CV) responses of the bare Au PCB and the rGO-Ag@SiO_2_/Au PCB in 0.1 M KCl. The calculated *C*_dl_ values of the bare Au and rGO-Ag@SiO_2_/Au PCBs were 1.58 and 2.20 mF/mm^2^, respectively (Figure S3c). These results show that the roughness factor of the Au PCB increased 1.4 times, owing to the modification of the Au PCB with the rGO-Ag@SiO_2_ nanocomposite. Evaluation of the ECASA values of the bare Au PCB and the rGO-Ag@SiO_2_–modified Au PCB was performed in the presence of 5 mM K_3_Fe(CN)_6_ in 0.1 M KCl. Figure S3d,e show the CV responses of the bare Au PCB and the rGO-Ag@SiO_2_–modified Au PCB. The Randles–Sevcik equation was applied to estimate the ECASA values [8]. The calculated ECASA values of the Au PCB and rGO-Ag@SiO_2_/Au PCB were found to be 2.210 and 13.812 mm^2^, respectively (Table S2). The percent real value (%) in terms of surface area is the ratio between ECASA and the geometric surface area of the Au PCB, which was found to be 122.3% [33,34].

**Figure 5:**
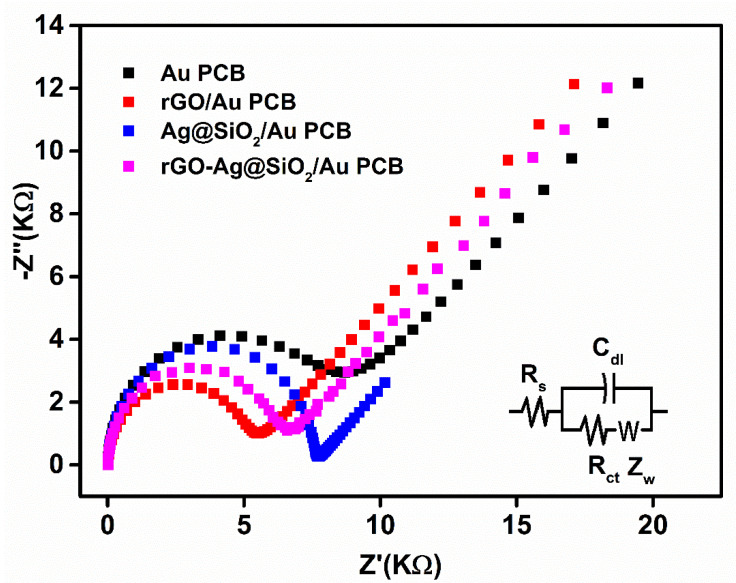
Electrochemical impedance spectroscopy (EIS) Nyquist plot of bare Au PCB, rGO/Au PCB, Ag@SiO_2_/Au PCB, and rGO-Ag@SiO_2_/Au PCB in 0.1 M KCl containing 1 mM K_3_Fe(CN)_6_.

In the presence of 5 mM K_3_Fe(CN)_6_, as a redox probe, electrochemical impedance spectroscopy (EIS) was used to characterize the rGO-Ag@SiO_2_–modified Au PCB electrodes in the frequency range between 0.1 mHz and 1 MHz. Higher frequencies could generate resistance, whereas diffusion was facilitated at lower frequencies in the electrodes. The bare Au PCB showed a higher charge transfer resistance (*R*_ct_) of 8.62 kΩ (Figure 5).

The rGO-modified Au PCB exhibited an excellent *R*_ct_ (5.53 KΩ) compared to the other modified electrodes in this study. The rapid electron transport of rGO caused fast charge transfer at the rGO/Au PCB electrode interface, which might be attributed to the low *R*_ct_ of the rGO/Au PCB. The Ag@SiO_2_/Au PCB demonstrated a high *R*_ct_ (7.73 KΩ) as a result of the generation of an electron blocking layer at the Ag@SiO_2_-modified Au PCB electrode. The formation of electronegative charges on the Ag@SiO_2_/Au PCB led to electrostatic repulsion between the Ag@SiO_2_/Au PCB and ferricyanide ions, which also generated the electron blocking layer. The rGO-Ag@SiO_2_–modified Au PCB generated a low *R*_ct_ (6.60 KΩ). The presence of rGO in rGO-Ag@SiO_2_ could be responsible for the enhanced charge transfer of the rGO-Ag@SiO_2_/Au PCB. This EIS study confirmed the importance of rGO in the rGO-Ag@SiO_2_ nanocomposite-modified Au PCB and that diffusion occurred at the rGO-Ag@SiO_2_/Au PCB.

### 3.3. Electrochemical Oxidation of EA in the rGO-Ag@SiO_2_/Au PCB

**Figure 6:**
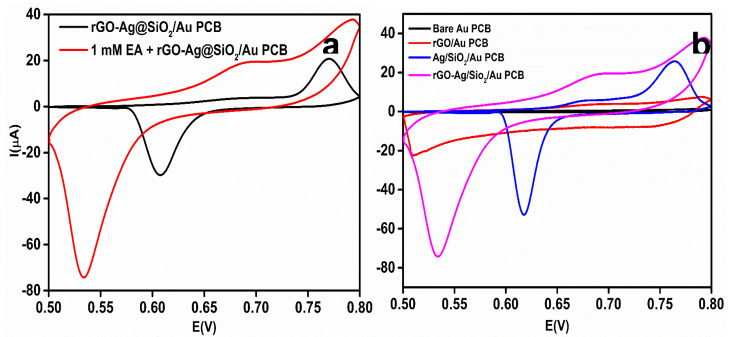
(**a**) Comparison of cyclic voltammetry (CV) response at rGO-Ag@SiO_2_/Au PCB in the absence and presence of 1 mM Etidronic acid (EA) in 0.1 M NaOH. (**b**) CV response of 1 mM EA at bare Au PCB, rGO/Au PCB, Ag@SiO_2_/Au PCB, and rGO-Ag@SiO_2_/Au PCB in 0.1 M NaOH.

To study the effect of EA on the rGO-Ag@SiO_2_/Au PCB, 1 mM EA in 0.1 M NaOH was used. To investigate the oxidation potential of EA and the electrocatalytic activity of the rGO-Ag@SiO_2_/Au PCB toward EA, CV responses in the presence and absence of EA were obtained (Figure 6a). After adding 1 mM EA, the current response at 0.67 V was increased to 19.4 μA. This shows the enhanced electrocatalytic ability and improved ECASA of the rGO-Ag@SiO_2_/Au PCB. The strengthening of the hydrogen bond between the SiO_2_ and the –OH group of EA might be the reason for the high selectivity toward EA as EA contains numerous hydroxyl groups in its structure.

The electrochemical oxidation of EA at the individual layers of the rGO-Ag@SiO_2_/Au PCB electrode was investigated. The bare Au PCB, rGO/Au PCB, Ag@SiO_2_/Au PCB, and rGO-Ag@SiO_2_/Au PCB electrodes were studied in the presence of 1 mM EA in 0.1 M NaOH electrolyte (pH = 13). Figure 6b shows the CV responses of the individual layers of the rGO-Ag@SiO_2_–modified Au PCB electrodes. The bare Au PCB failed to show a significant current response to EA. At 0.61 V, a very negligible current (0.25 μA) was obtained for EA. A significant increase in the current response (3.45 μA) was observed at 0.67 V for the rGO-modified Au PCB. The Ag@SiO_2_-modified Au PCB produced an enhanced current response (5.99 μA) at 0.68 V. Although the individual layer-modified Au PCB failed to show an elevated current response, the rGO-Ag@SiO_2_–modified Au PCB generated a current response of 19.4 μA, which was 77.6 times higher than that of the bare Au PCB. The higher current response for EA at the rGO-Ag@SiO_2_/Au PCB confirmed the improved electrocatalytic activity and enhanced electronic transport.

### 3.4. Effect of Scan Rate and pH

**Figure 7:**
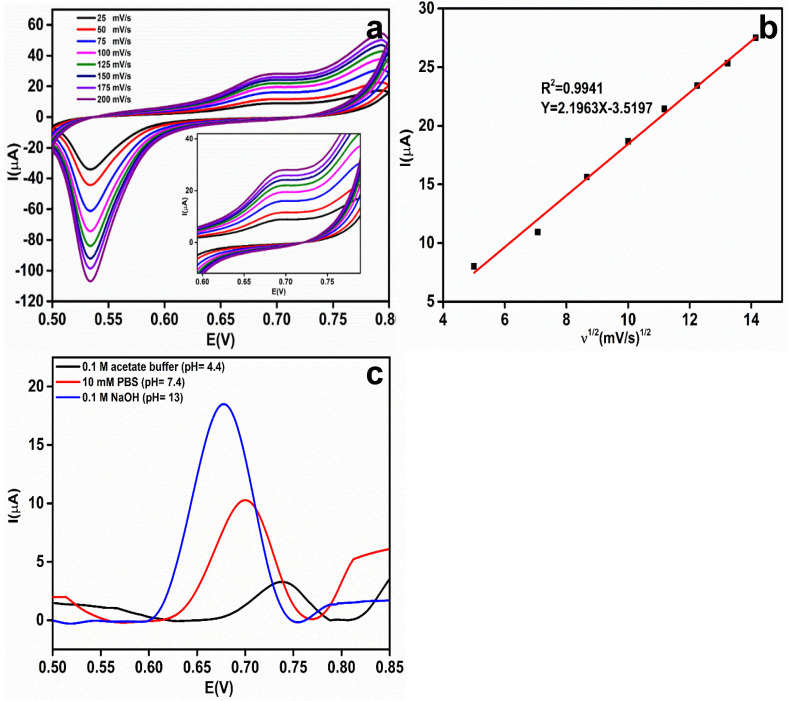
(**a**) Effect of the scan rate (25–200 mV/s) on the rGO-Ag@SiO_2_/Au PCB in the presence of 1 mM etidronic acid (EA) in a 0.1 M NaOH electrolyte (pH = 13). (**b**) Linear relationship between the square root of the scan rate (*ʋ*^1/2^) and the oxidation peak current (*I*_pa_) for EA. (**c**) Differential pulse voltammetric (DPV) response of 1 mM EA in different pH media [0.1 M acetate buffer (pH = 4.4), 10 mM PBS (pH = 7.4) and 0.1 M NaOH (pH = 13)].

To evaluate the effect of the scan rate on the electrochemical oxidation of 1 mM EA in 0.1 M NaOH, the CV responses were recorded at scan rates from 25 to 200 mV/s and the results are shown in Figure 7a. At 0.67 V, the anodic peak current corresponding to the oxidized EA increases linearly as the scan rate increases.

The Randles–Sevcik equation was applied to study the electrochemical oxidation process of EA at the rGO-Ag@SiO_2_–modified Au PCB: *I*_p_ = 2.69 × 10^5^ *n*^3/2^*AD*^1/2^*Cʋ*^1/2^, where *I*_p_ is the peak current of EA, *n* is the number of electrons participating in the reaction, *A* is the ECASA, *D* is the diffusion coefficient, *C* is the concentration of EA (mol/cm^3^), and *ʋ* is the scan rate (mV/s) [35]. The linear graph (Figure 7b) between the square root of the scan rate (*ʋ*^1/2^) and the corresponding anodic peak current of EA provides concrete confirmation that the electrochemical oxidation of EA is a diffusional process.

To enhance the stability and performance of the electrochemical sensor, a pH study was conducted in three different pH media containing 1 mM EA: 0.1 M acetate buffer (pH = 4.4), 10 mM PBS (pH = 7.4), and 0.1 M NaOH (pH = 13) [36]. It was observed (Figure 7c) that the oxidation potential (*E*_p_) shifted negatively with increasing pH of the electrolyte. A low current response (3.29 μA) was observed in the acidic medium. At neutral pH, an enhanced peak current (10.3 μA) was observed compared to the acidic medium. The highest peak current of EA (18.4 μA) was observed at 0.68 V in 0.1 M NaOH, and pH = 13.

### 3.5. Mechanism of the Detection of EA

**Scheme 1:**
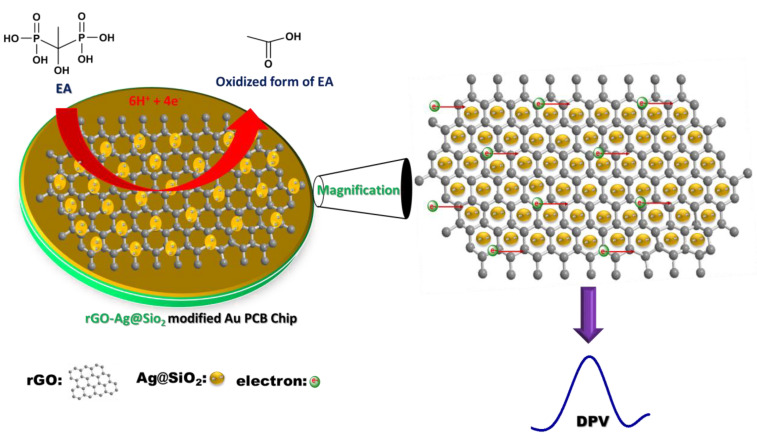
Mechanism in the electrochemical oxidation of etidronic acid (EA) at rGO-Ag@SiO_2_/Au PCB.

It is well known that primary and secondary alcohols may be oxidized directly to produce their corresponding aldehydes or ketones. However, tertiary alcohols can never be oxidized directly, thus, the electrochemically active functional groups for tertiary alcohols are oxidized. Hence, multiple steps are involved in the oxidation of electrochemically active functional groups in tertiary alcohols. EA followed a similar oxidation procedure (Figure S5). Initially, C–P bond breaking occurs via oxidation, which causes the formation of phosphate ions [37,38]. The oxidation process is driven by the participation of 1.5 times more protons than the number of electrons (3:2). The second step involves the removal of H_2_O from the intermediate product. The final step deals with the cleavage of C–P bonding and generates a phosphate ion by the participation of three protons and two electrons. Scheme 1 demonstrates how the rGO-Ag@SiO_2_–modified Au PCB facilitates improved electron transfer in the electrochemical detection of EA. The enhanced ECASA of the rGO-Ag@SiO_2_–modified Au PCB, in addition to the high number of active sites possessed by the rGO-Ag@SiO_2_ composite, could be attributed to the improved electrochemical detection of EA.

### 3.6. DPV Study

**Figure 8:**
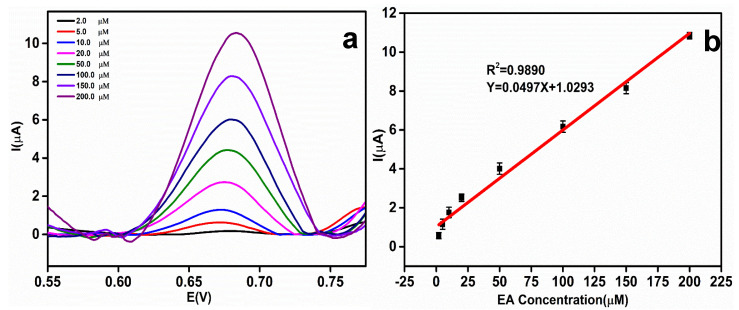
(**a**) Differential pulse voltammetric (DPV) response of etidronic acid (EA) at different concentrations (2.0, 5.0, 10.0, 20.0, 50.0, 100.0, 150.0, and 200.0 μM) in 0.1 M NaOH. (**b**) Linear relationship between the EA concentration and peak current.

Validation of the rGO-Ag@SiO_2_–modified electrodes at various concentrations of EA was performed using the DPV technique. As the concentration of EA increased, the oxidation peak current of EA increased. Figure 8a shows the DPV curves obtained with different concentrations of EA. The validated *E*_p_ of EA was obtained at 0.68 V. The *E*_p_ of EA remained constant and showed the greater stability of the rGO-Ag@SiO_2_–modified Au PCB electrodes. The EA concentration vs. oxidation peak current plot (Figure 8b) demonstrates that the oxidation peak current of EA increases linearly with increasing EA concentration. The linearity expression of EA concentration vs. EA peak current can be written as *Y* = 0.0497*X* + 1.0293 (R^2^ = 0.9890). The error bar signifies the standard deviation of the five determinants. To calculate the limit of detection (LOD) in the electrochemical detection of EA, the IUPAC method was used. As per this method, 10 blank injections of 0.1 M NaOH were added and the DPV responses were recorded. LOD = 3*S*_B_/*S* (*S*_B_: standard deviation of 10 blank samples, *S*: slope of the calibration curve) [39,40]. The LOD of the electrochemical detection of EA in the rGO-Ag@SiO_2_–modified Au PCB was calculated to be 0.68 μM. The constructed linear equation could be used to determine the unknown concentration of EA in the real sample analysis. The effectiveness of the rGO-Ag@SiO_2_/Au PCB-based EA sensor was compared with previously evaluated sensors, and the results are presented in Table S1.

### 3.7. Stability, Reproducibility, and Reusability

The operational stability of the rGO-Ag@SiO_2_/Au PCB electrochemical sensor was evaluated through CV in the presence of 1 mM EA in 0.1 M NaOH, pH = 13. Twenty CV cycles were recorded, and at the end of the 20th cycle, 92.7% of the initial peak current was retained. To assess the long-term stability of the rGO-Ag@SiO_2_/Au PCB in the detection of EA, the rGO-Ag@SiO_2_/Au PCB was stored at room temperature (25 °C), and a storage stability study was conducted at intervals of seven days for four weeks. At the end of the fourth week, 93.1% of the DPV peak current response of the initial peak current was retained. The evaluation of the reusability of the rGO-Ag@SiO_2_/Au PCB in the electrochemical detection of 100.0 μM EA was carried out using the DPV technique. A single rGO-Ag@SiO_2_/Au PCB was used continuously for up to eight runs. At the end of each run, the rGO-Ag@SiO_2_/Au PCB was cleaned with a 0.1 M NaOH buffer to eliminate the oxidized form of EA adsorbed on the PCB. The observed DPV current responses were plotted in a bar chart (Figure S6) to compare the eight runs. The evaluated percent relative standard deviation (%RSD) between the eight runs was calculated to be 3.51%. The reproducibility of the rGO-Ag@SiO_2_/Au PCB was investigated in the presence of 100.0 μM EA in 0.1 M NaOH using four independent rGO-Ag@SiO_2_/Au PCBs. The %RSD between the DPV current responses of EA was evaluated to be 2.88%. Table 1 shows the sensor parameters of rGO-Ag@SiO_2_ in EA detection.

### 3.8. Interference Study

**Figure 9:**
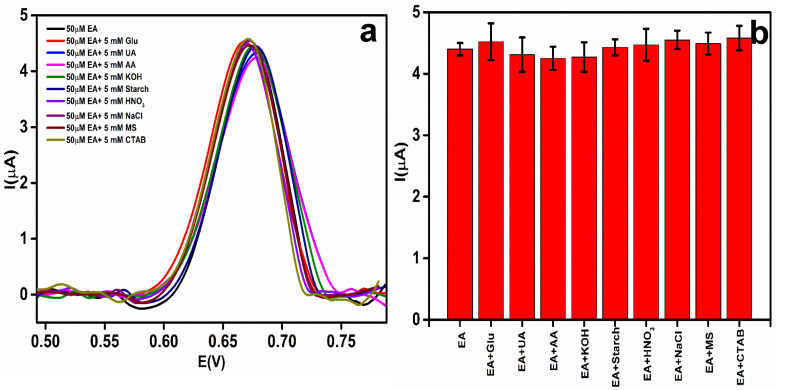
(**a**) Differential pulse voltammetric (DPV) response of an interferent study of etidronic acid (EA) (50 μM) with different interferents. (**b**) Bar chart of peak current response of EA with different interferents.

The selectivity of the EA detection at the rGO-Ag@SiO_2_/Au PCB was evaluated in the presence of other excipients, co-existing interferents, and ionic interferents. For the selectivity and to determine the effect of the interferents at the EA peak current and its *E*_p_, 50 μM EA was added to 5 mM each of different interferents: glucose (Glu), uric acid (UA), ascorbic acid (AA), potassium hydroxide (KOH), starch, nitric acid (HNO_3_), sodium chloride (NaCl), magnesium stearate (MS), and cetyl trimethyl ammonium bromide (CTAB) [41]. The recorded DPV responses are shown in Figure 9a. A comparison of the effect of different interferents on the EA peak current is displayed in Figure 9b.

Table 2 shows the current responses and the *E*_p_ values of EA after the addition of the different interferents. No significant change in the *E*_p_ of EA was observed. The change in the EA peak current owing to the addition of interferents was less than 5%. This proved that the developed sensor was highly selective toward EA.

### 3.9. Real Sample Analysis

**Figure 10:**
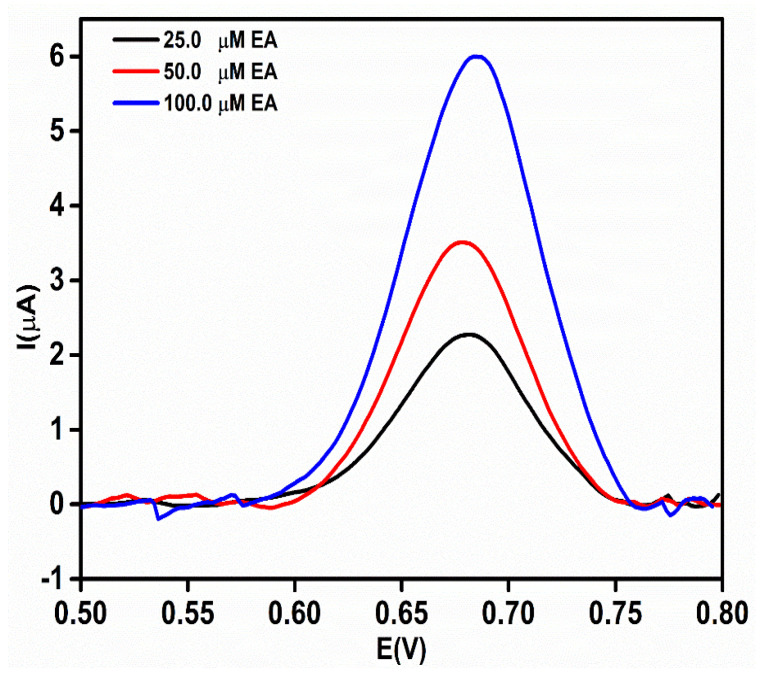
Differential pulse voltammetric (DPV) response of etidronate tablets at different concentrations (25.0, 50.0, and 100.0 μM) in 0.1 M NaOH.

To verify the real-time application of the proposed sensor, an rGO-Ag@Sio_2_–modified Au PCB-based electrochemical sensor was used to analyze the concentration of EA in etidronate tablets (25.0, 50.0, and 100.0 μM). The obtained DPV current responses (Figure 10) were fitted into the calibration curve (*Y* = 0.0497*X* + 1.0293) to calculate the actual concentration of the EA in the real sample. The recovery of EA in the pharmaceutical samples was calculated to be between 96.2% and 102.9% (Table S2).

## 4. Conclusions

Rapid construction of an rGO-Ag@SiO_2_/Au PCB-based electrochemical sensing platform was achieved through probe sonication. The self-assembled rGO-Ag@SiO_2_ nanocomposite was characterized by SEM, UV, FTIR, and XPS Steps involved in the electrochemical oxidation of EA were studied by CV and DPV. Electrochemical detection of EA in the rGO-Ag@SiO_2_/Au PCB was found to be a diffusion-controlled process with a linear range of 2.0–200.0 μM, and the obtained LOD was 0.68 μM. Using 100-fold higher concentrations of various interferent, detection of EA was carried out, and the effect of the various interferents at the EA peak current was less than 5%. The constructed rGO-Ag@SiO_2_/Au PCB-based DPV sensor exhibited excellent repeatability (%RSD = 3.51 for eight runs), high reproducibility (%RSD = 2.88 for four independent Au PCB electrodes), and long-term stability over four weeks. The rGO-Ag@SiO_2_/Au PCB sensor was tested on etidronate tablets, and the obtained recovery values were excellent (96.2–102.9%). Thus, the optimized rGO-Ag@SiO_2_/Au PCB electrochemical sensor could be used to detect EA in real samples.

## Figures and Tables

**Table 1:** Performance of rGO-Ag@SiO2/Au PCB sensor parameters.

Sensor Parameters	EA
Oxidation potential (V)	0.67
Linearity range (µM)	2.0–200.0
Slope of calibration curve	0.0497
Standard deviation of blanks	1.12 × 10^−8^
LOD (µM)	0.68
Operational stability (Retained peak current after 20 cycles)	92.7%
Repeatability (%RSD)	3.51%
Reproducibility (%RSD)	2.88%
Storage stability (Retained peak current after four weeks)	93.1%

**Table 2:** Interference study results in the electrochemical detection of EA.

Interferent Added	Concentration of Interferent Added (mM)	Concentration of EA Added (µM)	*E*_p_ of EA (V)	Peak Current of EA (µA)
-	0	50	0.675	4.43
Glucose	5	50	0.668	4.52
UA	5	50	0.679	4.31
AA	5	50	0.679	4.26
KOH	5	50	0.676	4.27
Starch	5	50	0.678	4.43
HNO_3_	5	50	0.670	4.47
NaCl	5	50	0.673	4.55
MS	5	50	0.673	4.49
CTAB	5	50	0.671	4.58

## Supplementary Materials

The following are available online at https://www.mdpi.com/2079-4991/10/7/1368/s1: Figure S1: Photographs of Au PCB; Figure S2: (a), (b), and (c) EDX spectra of bare Au PCB, rGO/Au PCB, and rGO-Ag@SiO_2_/Au PCB, respectively; Figure S3: (a), (b) CV responses of non-faradaic current response of bare Au PCB and rGO-Ag@SiO_2_/Au PCB, respectively, in 0.1 M KCl, (c) relationship between scan rate (ʋ) and current density, (d) and (e) CV response of bare Au PCB and rGO-Ag@SiO_2_/Au PCB in 0.1 M KCl containing 5 mM K3Fe(CN)6; Figure S4: (a) CV responses of rGO-Ag@SiO_2_ at various concentrations of rGO (0, 0.5, and 1.0 mg) and at a fixed concentration of Ag@SiO_2_ (1 mg) in the presence of EA in 0.1 M NaOH. (b) The plot of CrGO /CrGO + CAg@SiO_2_ vs. oxidation peak current (Ipa); Figure S5: Plausible steps involved in the electrochemical oxidation of EA; Figure S6: Eight consecutive DPV responses of 100.0 μM EA in 0.1 M NaOH at rGO-Ag@SiO_2_/Au PCB; Figure S7: Raman spectral analysis of graphite, GO, and rGO. Table S1: Comparison of previously reported materials in the detection of EA to the current study; Table S2: ECASA calculation; Table S3. Real-time analysis of EA in the pharmaceutical samples; Table S4. Raman spectral analysis of graphite, GO, and rGO.
