# Supplementary material for: Differential Pulse Voltammetric Electrochemical Sensor for the Detection of Etidronic Acid in Pharmaceutical Samples by Using rGO-Ag@SiO2/Au PCB
Source: Nanomaterials (Basel). 2020 Jul 14;10(7):1368. doi: 10.3390/nano10071368 (PMC7407910; doi:10.3390/nano10071368)
Supplement: Supplementary file 1 [file nanomaterials-10-01368-s001.pdf]

Differential Pulse Voltammetric Electrochemical Sensor for the Detection of Etidronic Acid in Pharmaceutical Samples by using rGO-Ag@SiO$_2$/Au PCB

Sathish Panneer Selvam [1], Somasekhar R. Chinnadayyala [1], Sungbo Cho [1,2,*], and Kyusik Yun [3,*]

[1] Department of Electronics Engineering, Gachon University, Seongnam-si, Gyeonggi-do 13210, Korea; satp103@gc.gachon.ac.kr (S.P.S.); ssreddy@gachon.ac.kr (S.R.C.)
[2] Gachon Advanced Institute for Health Science & Technology, Gachon University, Incheon 21999, Korea
[3] Department of Bionanotechnology, Gachon University, Seongnam-si, Gyeonggi-do 13210, Korea
* Correspondence: sbcho@gachon.ac.kr (S.C.); ykyusik@gachon.ac.kr (K.Y.)

Chemicals and apparatus

Tetraethyl orthosilicate (TEOS) [$Si(OC_2H_5)_4$], silver nitrate ($AgNO_3$), ammonium hydroxide (NH_4OH), sodium borohydride ($NaBH_4$), sodium hydroxide (NaOH), sulfuric acid (H_2SO_4), glucose (Glu), ascorbic acid (AA), sodium chloride (NaCl), L-dopamine hydrochloride (DA), potassium ferricyanide [$K_3Fe(CN)_6$], potassium chloride (KCl), cetyl trimethyl ammonium bromide (CTAB), starch, hydrochloric acid (HCl), nitric acid (HNO_3), magnesium stearate (MS), and 10 mM phosphate-buffered saline (PBS) were purchased from Sigma Aldrich, Korea. An alumina slurry (0.05 µm size) was procured from ALS Co., Ltd., Japan. Sodium acetate was purchased from Junsei Chemical Co., Ltd. Hydrochloric acid (HCl), potassium permanganate ($KMnO_4$), and hydrogen peroxide (H_2O_2) were purchased from DAEJUNG Chemicals & Metals Co., Ltd, Korea. Milli-Q water was used throughout the analysis. Etidronate tablets (200 mg) were procured from Mylan, India.

The morphology of rGO-Ag@SiO$_2$ was characterized by scanning electron microscopy (SEM) [JEOL (Japan) at 15 kV acceleration voltage with energy dispersive X-ray (EDX)] and transmission electron microscopy (FEI Titan 80-300, OR, USA). To analyze the ultraviolet-visible (UV-vis) spectrum, a Shimadzu UV-vis spectrophotometer (UV-2550) with 1-cm quartz cells was used. Fourier-transform infrared (FTIR) spectroscopy using a Thermo Nicolet iS10 spectrometer (Korea) was used to study the chemical properties of rGO–Ag@SiO$_2$ and KBr (Sigma Aldrich, Korea) was used to make pellets during FTIR analysis. The presence of C, O, Ag, and Si in rGO-Ag@SiO$_2$ and its electronic structure were confirmed by X-ray photoelectron spectroscopy (XPS) [PHI 5000 Versa Probe (Ulvac-PHI) spectrometer (Japan) with monochromator A1 Kα (1486.6 eV)]. Electrochemical experiments were carried out using a PARSTAT 2273 Advanced Electrochemical System (Princeton Applied Research, TN, USA). An Au PCB, which consisted of a working electrode (Au), reference electrode (Au), and counter electrode (Au), was used for the electrochemical analysis. pH testing was performed using a Thermo Fisher Scientific pH meter (Orion star A210, MA, USA). A SONIC VCX 750 (CT, USA) model with a titanium probe was used for ultrasound irradiation. A UniDRON (Korea) system was used to study the Raman spectra of graphite, GO, and rGO with a 532 nm laser.

Electrochemical active surface area (ECASA) study

R_f was calculated from the double-layer capacitance (C_{dl}). The non-faradaic current was captured (Figures S3a and S3b) at different scan rates (25 to 200 mV/s) with a potential window from −0.7 to −0.2 V. The current density at −0.45 V was considered to construct the C_{dl} (Figure S3c). The following expressions were used to calculate R_f:

$$R_f = C_{dl}/40\ \mu F \quad (1)$$

The S-geometric surface area of the bare Au PCB was 0.0179 cm^2.

Evaluation of the electrochemical active surface areas of the Au PCB and rGO-Ag@SiO_2–modified Au PCB electrodes were evaluated using the Randles–Sevcik equation.

$$I_{pa} = 2.69 \times 10^5 \, n^{3/2} A D^{1/2} C v^{1/2} \quad (2)$$

where I_{pa} = anodic peak current, n = number of electrons transferred, A = ECASA, D = diffusion coefficient (D = 7.6 × 10^{-6} cm^2/s), C is the concentration of potassium ferricyanide (mol/cm^3), and v = scan rate (mV/s). The CV responses of the bare Au PCB and the rGO-Ag@SiO_2/Au PCB are shown in Figures S3d and S3e, respectively. The evaluated ECASAs for the bare Au PCB and the rGO-Ag@SiO_2/Au PCB were 2.210 and 13.812 mm^2, respectively.

Optimization of the rGO and Ag@SiO_2 quantities

Optimization of rGO and Ag@SiO_2 was carried out by fixing the concentration of Ag@SiO_2 at 1 mg mL^{-1}, whereas the concentration of rGO was increased from 0 to 1 mg mL^{-1} (0, 0.5, and 1.0 mg mL^{-1}). The optimization of the loading quantity of rGO and Ag@SiO_2 was investigated through CV in 0.1 M NaOH containing 1 mM EA. The CV responses of different concentrations of rGO-Ag@SiO_2 showed that as the concentration of rGO increased from 0 to 1 mg mL^{-1}, the anodic peak current was enhanced to 19.4 μA (Figures S4a and S4b). This confirms the importance of rGO in enhancing the electrocatalytic activity in the electrochemical detection of EA.

Raman spectroscopic study of graphite, GO, and rGO

Raman spectral analysis is the most frequently used spectroscopic method to characterize carbon-based nanomaterials such as graphene and its derivatives. Two characteristic peaks were recorded for graphite, GO, and rGO, namely, D (defect in graphene) and G (symmetrical peak) bands and a weak 2D band. Figure S7 depicts the Raman spectral analysis of graphite, GO, and rGO. The I_D/I_G values provide information about the disorder in graphene and can be used to distinguish GO from rGO. I_D/I_G may increase or decrease as GO is converted to rGO. The intensities of the corresponding Raman shift values for graphite, GO, and rGO are presented in Table S4. In graphite, weak D (1330.5 cm^{-1}), intense G (1560.1 cm^{-1}), and 2D (2679.3 cm^{-1}) peaks were observed. GO showed intense D and G peaks and a weak 2D peak at 1340.9, 1573.5, and 2654.3 cm^{-1}, respectively. Finally, in rGO, high-intensity D (1348.4 cm^{-1}) and G (1585.4 cm^{-1}) peaks were observed, along with a very weak 2D peak (2670.9 cm^{-1}). An increase in I_D/I_G strongly suggests a high defect density in GO and rGO. rGO possesses a high I_D/I_G value compared to graphite and GO, which confirms the higher number of nanocrystalline aromatic carbons [1].

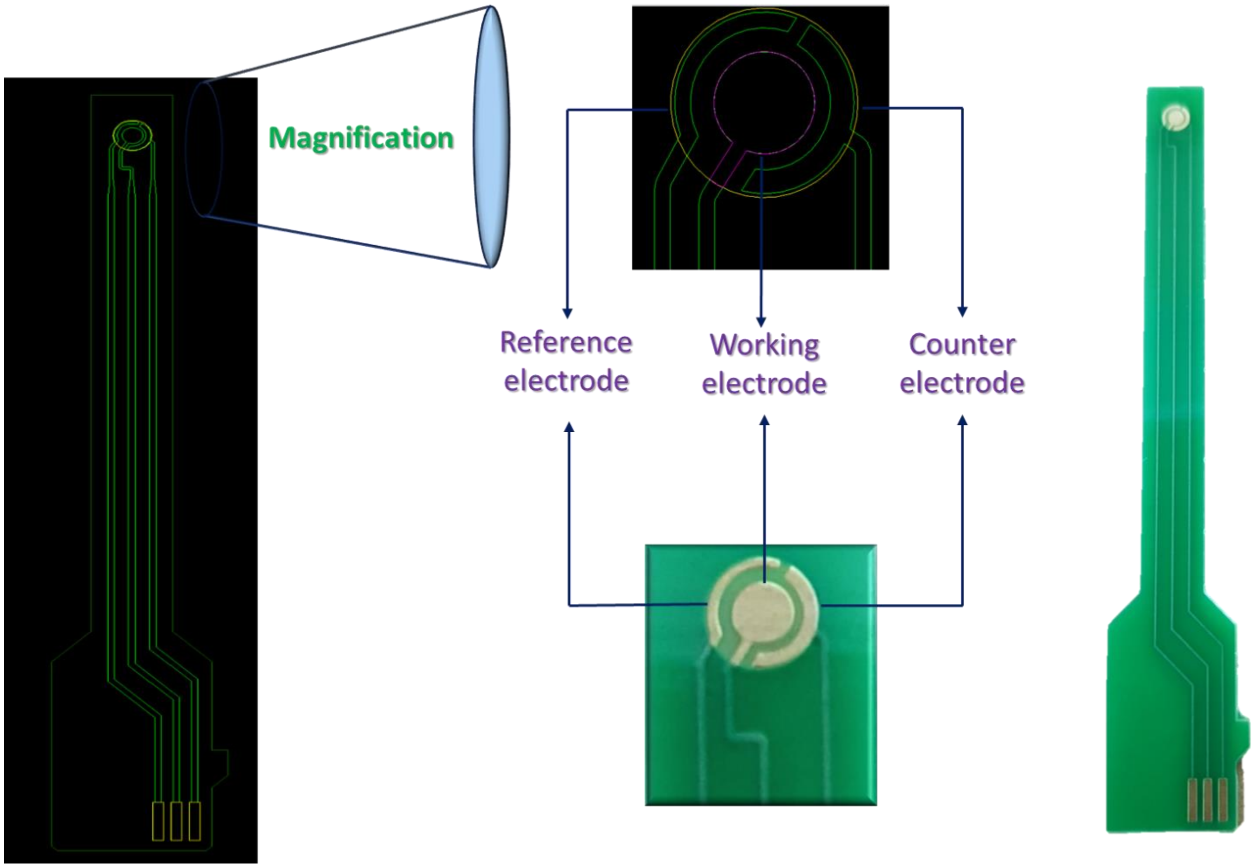

Figure S1. Photographs of Au PCB.

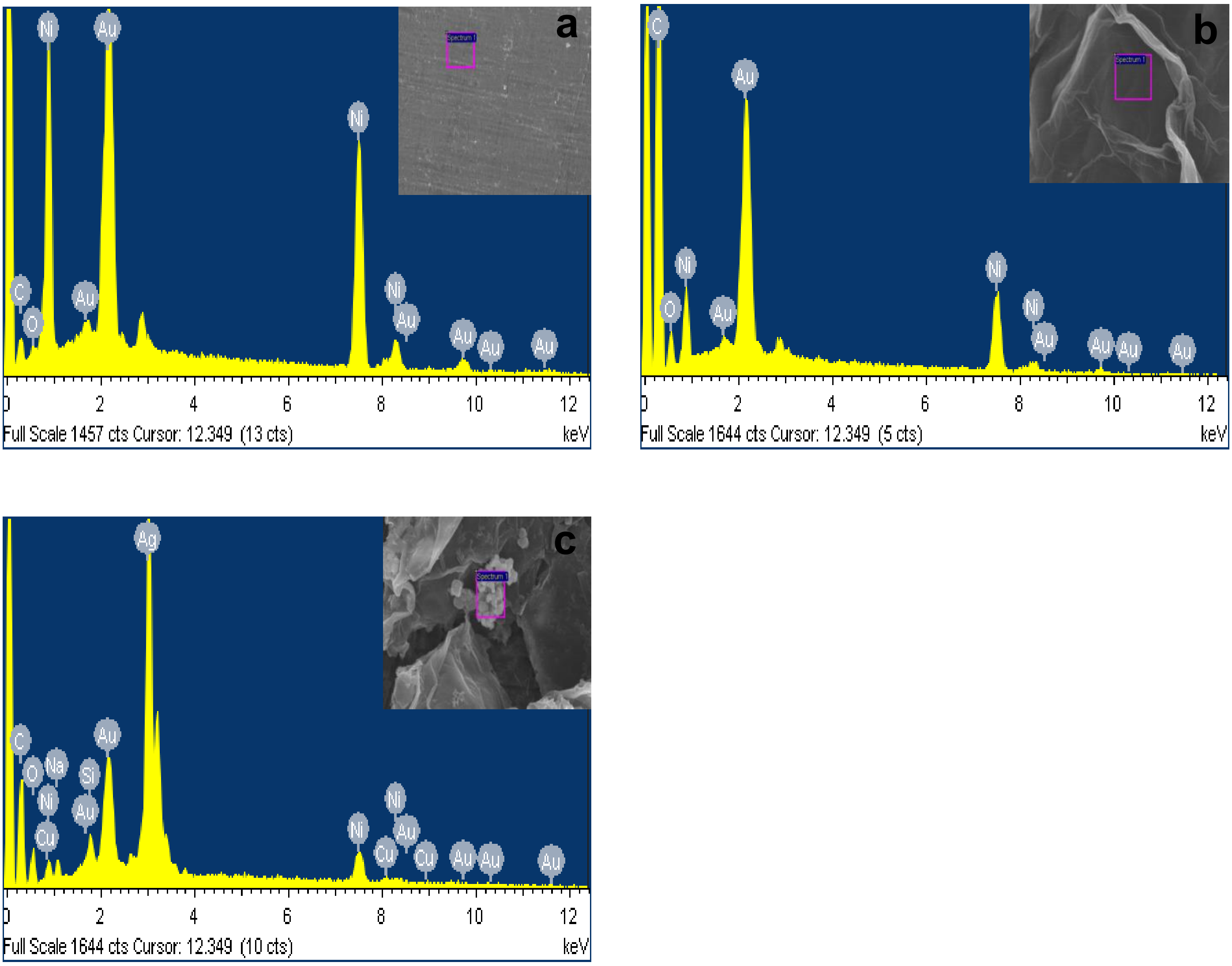

Figure S2. (**a**), (**b**), and (**c**) EDX spectra of bare Au PCB, rGO/Au PCB, and rGO-Ag@SiO_2/Au PCB, respectively.

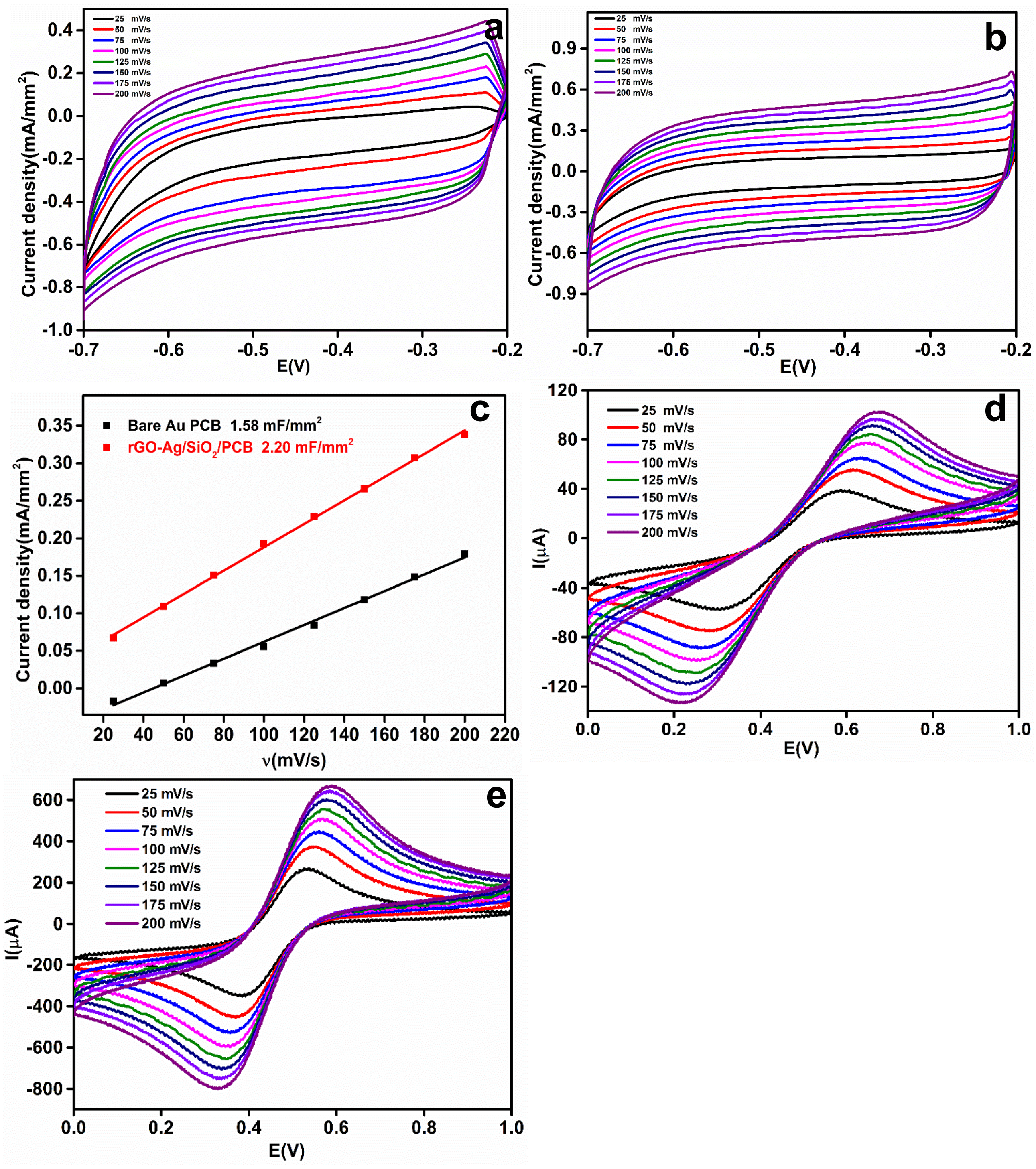

Figure S3. (**a**), (**b**) CV responses of non-faradaic current response of bare Au PCB and rGO-Ag@SiO_2/Au PCB, respectively, in 0.1 M KCl. (**c**) relationship between scan rate (*v*) and current density, (**d**) and (**e**) CV response of bare Au PCB and rGO-Ag@SiO_2/Au PCB in 0.1 M KCl containing 5 mM $K_3Fe(CN)_6$.

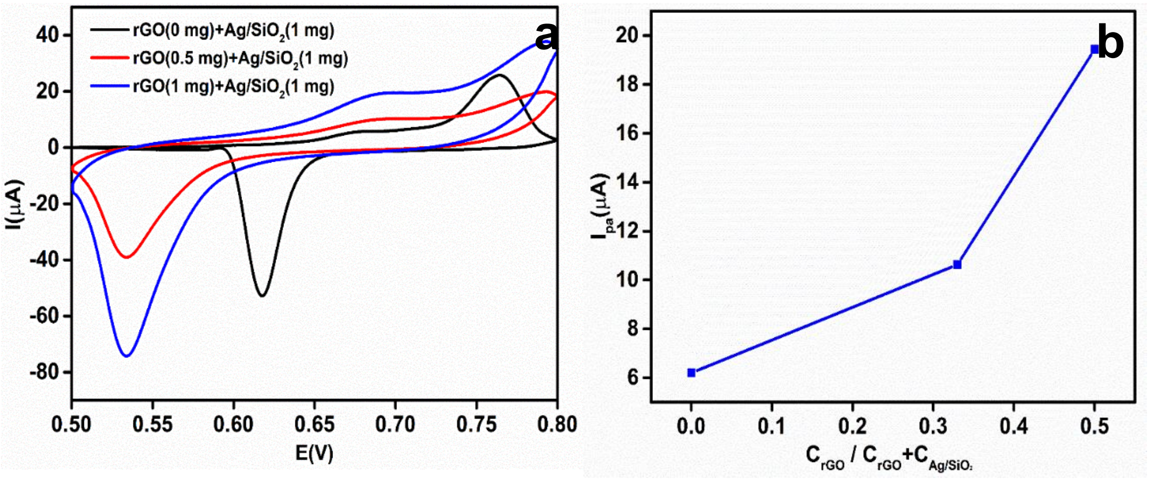

Figure S4. (**a**) CV responses of rGO-Ag@SiO2 at various concentrations of rGO (0, 0.5, and 1.0 mg) and at a fixed concentration of Ag@SiO2 (1 mg) in the presence of EA in 0.1 M NaOH. (**b**) The plot of C_{rGO} /C_{rGO} + $C_{Ag@SiO2}$ vs. oxidation peak current (I_{pa}).

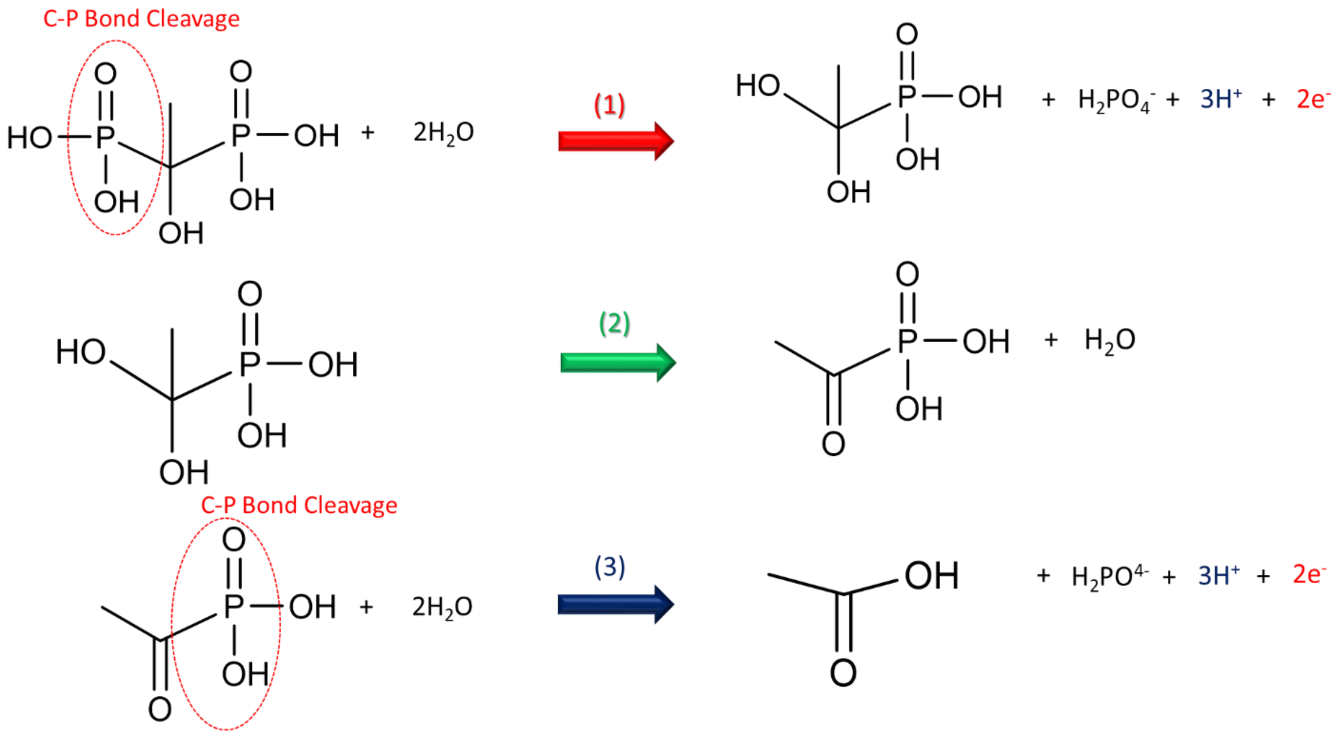

Figure S5. Plausible steps involved in the electrochemical oxidation of EA.

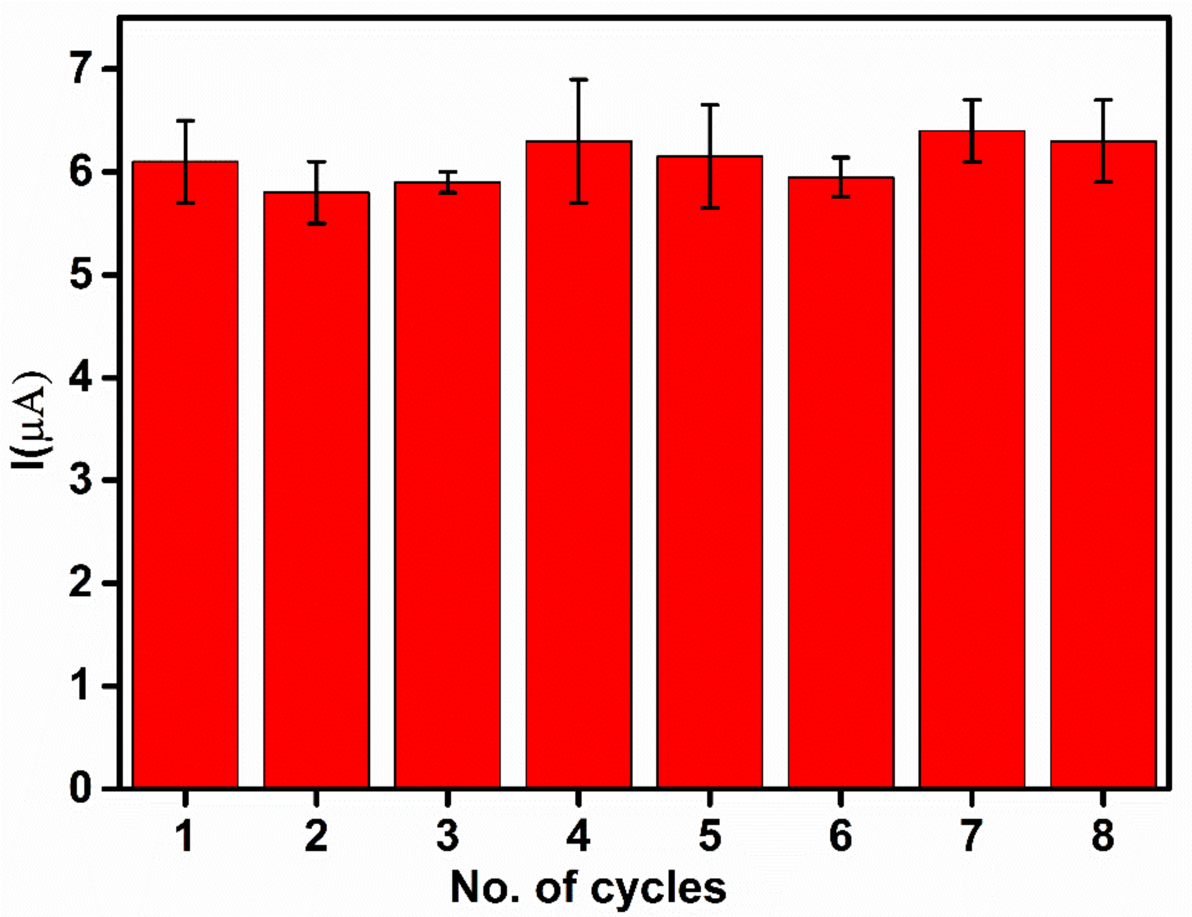

Figure S6. Eight consecutive DPV responses of 100.0μM EA in 0.1 M NaOH at rGO-Ag@SiO_2/Au PCB.

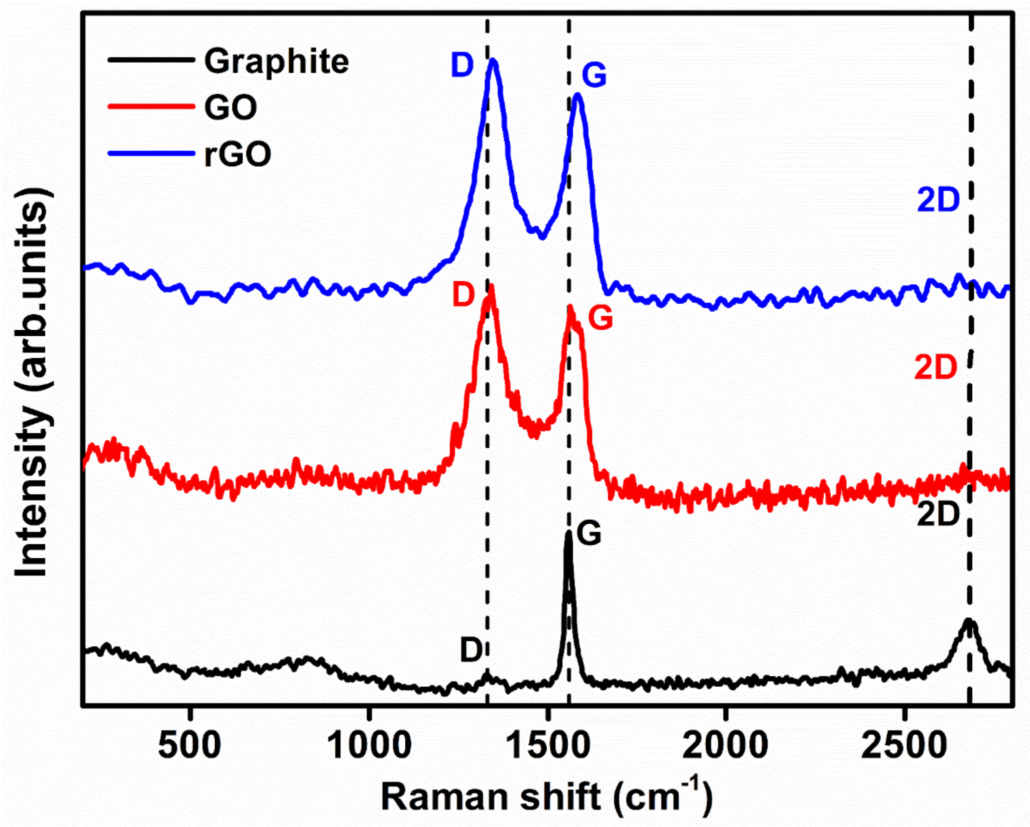

Figure S7. Raman spectral analysis of graphite, GO, and rGO.

Tables

Table 1. Comparison of previously reported materials in the detection of EA to the current study.

Method of Detection	Linear Range (μM)	LOD (μM)	Ref.
Spectrophotometeric determination	8–96	3.2	[2]
Ion chromatography with indirect UV detection	264–2640	4.0	[3]
Amperometry using copper nanoparticles-modified carbon paste electrode	200–2538	10.6	[4]
Mixed-mode column chromatography	5000–30000	40.0	[5]
Reverse-phase chromatography	150–2400	72.8	[6]
DPV sensor based on rGO-Ag@SiO_2–modified Au PCB	2–200	0.68	This work

Table 2. ECASA calculation.

Redox Probe	Added	ECASA (mm^2)
$K_3Fe(CN)_6$	Au PCB	2.210
	rGO-Ag@SiO_2/Au PCB	13.812

Table 3. Real-time analysis of EA in the pharmaceutical samples.

	Added	Found	R.S.D. (%)	Recovery (%)
Etidronate tablet	25.0 μM	25.7 μM	2.39	102.9
	50.0 μM	49.2 μM	1.43	98.3
	100.0 μM	99.2 μM	0.86	99.2

Table S4. Raman spectral analysis of graphite, GO, and rGO.

Material	Raman Shift			Intensity			I_D/I_G
	D	G	2D	D	G	2D	
**Graphite**	1330.5	1560.1	2679.3	269.3	351.6	391.1	0.77
**GO**	1340.9	1573.5	2654.3	386.2	374.7	375.8	1.03
**rGO**	1348.4	1585.4	2670.9	423.5	402.9	365.4	1.05

Reference

1. Aunkor, M.T.H.; Mahbubul, I.M.; Saidur, R.; Metselaar, H.S.C. The green reduction of graphene oxide. *RSC Adv.* **2016**, *6*, 27807–27825, doi:10.1039/c6ra03189g.
2. Taha, E.A.; Youssef, N.F. Spectrophotometric determination of some drugs for osteoporosis. *Chem. Pharm. Bull.* **2003**, *51*, 1444–1447, doi:10.1248/cpb.51.1444.
3. Tsai, E.W.; Ip, D.P.; Brooks, M.A. Determination of etidronate disodium tablets by ion chromatography with indirect UV detection. *J. Pharm. Biomed. Anal.* **1993**, *11*, 513–516, doi:10.1016/0731-7085(93)80165-W.
4. Heli, H.; Faramarzi, F.; Jabbari, A.; Parsaei, A.; Moosavi-Movahedi, A.A. Electrooxidation and determination of etidronate using copper nanoparticles and microparticles-modified carbon paste electrodes. *J. Braz. Chem. Soc.* **2010**, *21*, 16–24, doi:10.1590/S0103-50532010000100004.
5. Liu, X.K.; Fang, J.B.; Cauchon, N.; Zhou, P. Direct stability-indicating method development and validation for analysis of etidronate disodium using a mixed-mode column and charged aerosol detector. *J. Pharm. Biomed. Anal.* **2008**, *46*, 639–644, doi:10.1016/j.jpba.2007.11.041.
6. Xie, Z.; Jiang, Y.; Zhang, D.Q. Simple analysis of four bisphosphonates simultaneously by reverse phase liquid chromatography using n-amylamine as volatile ion-pairing agent. *J. Chromatogr. A* **2006**, *1104*, 173–178, doi:10.1016/j.chroma.2005.11.113.
